# Exploring the Relationship Between Online Social Network Site Usage and the Impact on Quality of Life for Older and Younger Users: An Interaction Analysis

**DOI:** 10.2196/jmir.5377

**Published:** 2016-09-29

**Authors:** Darren Quinn, Liming Chen, Maurice D Mulvenna, Raymond Bond

**Affiliations:** ^1^ Computer Science Research Institute Faculty of Computing and Engineering Ulster University Newtownabbey United Kingdom; ^2^ De Montfort University School of Computer Science and Informatics Leicester United Kingdom

**Keywords:** online social network, social networking, Facebook, quality of life, interaction analysis, younger users, older users

## Abstract

**Background:**

Analyzing content generated by users of social network sites has been shown to be beneficial across a number of disciplines. Such analysis has revealed the precise behavior of users that details their distinct patterns of engagement. An issue is evident whereby without direct engagement with end users, the reasoning for anomalies can only be the subject of conjecture. Furthermore, the impact of engaging in social network sites on quality of life is an area which has received little attention. Of particular interest is the impact of online social networking on older users, which is a demographic that is specifically vulnerable to social isolation. A review of the literature reveals a lack of knowledge concerning the impact of these technologies on such users and even less is known regarding how this impact varies across different demographics.

**Objective:**

The objective of our study was to analyze user interactions and to survey the attitudes of social network users directly, capturing data in four key areas: (1) functional usage, (2) behavioral patterns, (3) technology, and (4) quality of life.

**Methods:**

An online survey was constructed, comprising 32 questions. Each question directly related to a research question. Respondents were recruited through a variety of methods including email campaigns, Facebook advertisements, and promotion from related organizations.

**Results:**

In total, data was collected from 919 users containing 446 younger and 473 older users. In comparison to younger users, a greater proportion of older users (289/473, 61.1% older vs 218/446, 48.9% younger) (*P*<.001) stated that Facebook had either a positive or huge impact on their quality of life. Furthermore, a greater percentage of older users strongly agreed that Facebook strengthened their relationship with other people (64/473, 13.5% older vs 40/446, 9.0%younger) (*P*=.02). In comparison to younger users, a greater proportion of older users had more positive emotions—classified as slightly better or very good—during their engagement with Facebook (186/473, 39.3% older vs 120/446, 26.9% younger) (*P*<.001).

**Conclusions:**

The results reveal that despite engaging at considerably lower rates with significantly fewer connections, older users gain a greater quality-of-life benefit. Results disclose how both cohorts vary in their use, interactions, and rationale for engaging with Facebook.

## Introduction

### Background

The benefits of analyzing user interactions generated from social network sites (SNS) have been well-documented [1-6]. A key finding has been the ability to isolate and report on not just individual behaviors, but also on specific user groups that can be attributed to specific patterns of engagement among variant age cohorts [7,8]. This paper looks to add to the body of evidence that younger and older users interact differently with feature usage and engagement frequency. In addition, we will begin to address if SNS have an impact on the quality of life (QoL) of their users. QoL is understood as being an “individual’s perception of their position in life in the context of the culture and value systems in which they live and in relation to their goals, expectations, standards and concerns” as defined by the World Health Organization [9].

Applying this view on QoL, through several works it is clear that social interaction is a key aspect for individuals; however, it is particularly so for older people [10-15]. Evidence reveals that declines in QoL directly relate to the vulnerability felt by older people from social isolation and loneliness [10,11,16-18]. The percentage of the world’s older population (ie, 60+ years of age) will increase from 12.3% to 21.5% in 2050 [19]. SNS are an exciting option when we consider potential solutions that have the ability to impact large-scale populations and combat social isolation for the elderly. However, at present little is known about how using SNS impacts the QoL of its users.

Given that younger users are accepted as having much greater volumes of online social activity [7,20-22], does this necessarily imply that SNS have a greater impact upon a younger user’s QoL? Or could the reverse be true, that older users with lower volumes of activity to have a higher QoL, with a focus on quality, not quantity, of SNS interactions? Important questions relating to the relationship between SNS usage and QoL remain unanswered. This is largely due to the complexities in the dual process of acquiring SNS user-generated content (UGC) for divergent age groups and then measuring the QoL of both cohorts. However, if achieved, the result would detail what relationships exist between age, usage, and QoL.

### Related Work

A review of literature detailing how users perceive the impact of SNS on QoL has been carried out; it details how the views of network users have been assessed to date. Burke et al [23] provided an analysis of the relationship between usage of the popular social network site Facebook and social well-being, whereby 1193 users were recruited via a Facebook ad campaign. Crucially, it investigated the concept of social capital (ie, networks forming together for mutual benefit) in the context of using Facebook, which had been observed in similar works [24,25]. Importantly, however, this work was largely apprehensive of findings by related works [25-28]. Burke et al [23] investigated if findings in related research [25-28] showed whether students who are active do experience higher levels of social capital and whether this can be generalized. The methodology employed a study into the precise interactions to which social capital can be attributed (eg, wall posts and friends’ conversations with other friends). Resulting outcomes demonstrated that directed interaction (eg, a wall post) is akin to greater feelings of bonding and lower levels of loneliness. Results confirmed that the use of SNS increases social capital and reduces loneliness, stating that “engagement with Facebook is correlated with greater overall well-being.”

There are a number of further works associating well-being and a user’s online interactions [29-34]. Sundar et al [35] explored the issue with regard to older users. Their work focused on evaluating usage by retirees on Facebook, asking if SNS can help alleviate social isolation from aging alone, whereby the QoL of subjects was measured through 33 items adapted using the Life Satisfaction Index (LSI). The work focused on the vulnerability of older users in assessing the potential of SNS, particularly that of Facebook, to positively contribute to the lives of retirees. Results stated that QoL was “not linked to Facebook use, frequency of use, or intensity.” However, this fact was stressed as being likely due to a number of factors. First, it observed users as having a small number of online friends and the small amount of time spent on Facebook by each subject. Second, a limitation was that only 34 retired Facebook users were assessed. Moreover, it was observed that the sample of older users already had a high QoL rating, leaving little room for any impact to be observed.

As highlighted by Burke et al [23], an issue within the literature at the time of this study is that studies are largely restricted to observing college students or adolescents only. Sundar et al [35] stress the significant fact that older users are distinct in nature from their younger counterparts (eg, lifestyles and experiences). Moreover, the work states that younger users have varying motivations for engaging with such technologies. It is clear that previous approaches were not explicit and direct in evaluating the experiences of users and the impact these experiences had on their QoL. For example, users were not directly asked if using application “x” or feature “x” contributed to their overall QoL and, if so, to what extent. As a result, this study aims to address these issues by constructing a questionnaire capable of evaluating user experience regardless of age or gender, an approach which can be employed for cross-comparison among varying demographics.

In summary, the literature shows that SNS can have a direct positive impact upon the well-being of users. However, the literature suggests that critical knowledge gaps remain in understanding the impact they have on the well-being of older users, since few works observed older users as a discrete cohort and, more crucially, they have not yet been directly compared and contrasted with a younger cohort.

### Study Aim

Since SNS can *potentially* alleviate the burden of social isolation for older users, the extent of this impact upon end users’ needs was assessed and quantified. The findings would have many applications, from formal policy decisions to design and usability considerations.

As social interaction is a key contributor to QoL [14], the following presented work investigated the impact of interacting within Facebook upon the QoL of two distinct age cohorts: those considered *young*, chosen for being the primary user of the technology, versus an *older* cohort, a grouping which may be vulnerable to increasing levels of social isolation and may therefore benefit from a technology designed to increase social interactivity.

In the context of this study and keeping in line with several previous works [5,7,8,20,36] in this area, users between 15 and 30 years of age were labeled as *younger* and those aged 50+ years old were labeled as *older*. This work extends current knowledge by examining the perceptions of users as they engage with SNS. It establishes relationships, if any, between SNS and the individual user’s QoL. We hypothesized that, given that interaction and usage have been shown to differ between older and younger users, we would expect SNS to positively contribute to the QoL of older people.

## Methods

### Overview

Within this section we present the methods applied for identifying and engaging with end users, which principally involved the development of an online survey. We also discuss the methods employed in data gathering and analysis.

### Data Collection

The design of the survey was driven by a series of 18 research questions (see Multimedia Appendix 1) based on several previous studies [5,7,8]. This survey aimed to capture the views of both cohorts in four key areas: (1) functional usage, (2) behavioral patterns, (3) technology, and (4) QoL impact. The approach to the phrasing of questions was that of a nontechnical, simplistic approach, being as concise as possible throughout. The online survey comprised 32 questions as presented in Table 1. The survey was implemented and hosted using SurveyGizmo (Widgix, LLC) [37].

### Survey Testing

Following the preliminary design, testing was carried out with a range of subjects who varied in terms of both gender and age. The aim of testing was to acquire user feedback in terms of design and coherence for end users. It requested feedback on aesthetics, question style, and phraseology, as well as overall usability. A series of survey iterations occurred following test responses, in advance of an agreed-upon final version.

Given that this research was to involve surveying two distinct age cohorts, an approach was applied to employ separate surveys. Given the depth of literature on barriers for older users, it was viewed that a one-look survey would be unlikely to be satisfactory for both cohorts. Older users were provided with minimal text, with only critical information to minimize cognitive strain, with a larger font size to increase readability. However, for both cohorts every question in each section (from Demographics section onwards) was identical and compulsory, regardless. To reduce survey dropouts, an error message indicated incomplete questions acting as a control loop. A progress bar was also provided to indicate progression.

### Sample Size

Required user numbers were determined through sample-size calculations. A confidence level of 95% was applied. At the time of data collection, the UK Facebook population was 28,940,400, with calculations determining the required sample size to be 474 completed surveys [8]. Consideration was given to the volumes of users recruited within related research. However, as previously noted, only a limited number of works were available, providing a lack of consistency in relation to user numbers in this area. Nevertheless, it is observed in the work of Sundar et al [35] that issues were raised concerning the ability to determine a QoL impact following the evaluation of only a small number of individuals (ie, n=34). Although no older users were sampled, an approach more akin to that of Burke et al [23] (ie, with significantly higher numbers [n=1193]) was viewed as providing greater confidence in terms of reliability and representative analysis.

### Recruitment

As a first phase uptake of younger users, an email was circulated to all Ulster University students in April 2012, with an estimated reach of approximately 28,500 registered students. A secondary phase consisted of posting on a series of Facebook accounts to stimulate interest and uptake, along with the use of Twitter to publicize the survey. The application of an all-student email proved extremely successful, quickly acquiring more than the required number of users. Recruitment of older users proved significantly more challenging. First, organizations with direct access to potential older subjects were consulted (see Multimedia Appendix 2) and asked for support in publicizing through their media outlets (eg, websites, blogs, Facebook, and Twitter accounts). A secondary phase promoted the survey during the annual *National Silver Surfer Day 2012*, an event aimed at encouraging the over 50-year-olds to engage with online technologies. In a localized context, the event was promoted in Northern Ireland libraries that hosted open days. Promotional leaflets were distributed throughout centers, providing information and a link to the study to encourage local uptake. Both strategies proved unfruitful. A final phase of Facebook advertising was employed as the core promotional strategy. This was a direct approach to engage end users, enabling advertisements to be displayed on the walls of a highly specific demographic. For this directly targeted audience, advertisements were displayed on only those profiles of Facebook users who were (1) over 50 years of age and (2) had attended university. The result was that two groups were acquired with a comparable socioeconomic status, with knowledge that both populations had entered third-level education with similar education attainment. Furthermore, with knowledge of the groups’ educational attainment, it provided an indication of both groups sharing a similar socioeconomic status.

**Table 1:** Online survey questions and answer styles.

Category		Question	Answer style
**Demographics**			
1		Gender	Radio buttons
2		Age	Textbox
3		Which of the following do you currently, or have you ever attended?	Radio buttons
4		How would you rate your level of computer literacy?	Likert scale
5		Please indicate which of the following Online Social Networks (OSN) accounts you have.	Check buttons
6		Do you have a Facebook account?	Radio buttons
**Functional usage**			
7		How important is Facebook for maintaining your real-world social connections?	Likert scale
8		How much consideration goes into adding or accepting new friend connections?	Likert scale
9		A list of Facebook’s most popular features has been compiled. Please rate each function in terms of importance.	Table of radio buttons
10		How important do you feel it is to keep your Facebook profile up-to-date, such as changing your profile picture or updating your relationship status, etc?	Likert scale
11		In relation to the above question, please take a moment to state why.	Textbox
12		After you created your Facebook account, rate how easy or difficult you found it to use its applications/functions.	Likert scale
13		In relation to the above question, please take a moment to state why.	Textbox
**Patterns of usage**			
14		How often do you log into Facebook?	Radio buttons
15		Indicate which you feel is your prime time for activity.	Radio buttons
16		Which day are you most likely to check Facebook for updates?	Radio buttons
17		Which day are you most likely to use Facebook for making plans (eg, social events)?	Radio buttons
18		Do you feel using Facebook is part of your routine?	Radio buttons
19		How important do you feel Facebook is for planning and broadcasting new events?	Likert scale
20		From the three options, rank how you would describe your general behavior when using Facebook.	Rank 1^st^, 2^nd^, and 3^rd^
21		Generally speaking, how much thought would you put into the posting of comments or replies?	Likert scale
22		Do you use Facebook more during the week or at the weekend?	Radio buttons
23		In relation to the above question, please take a moment to state why.	Textbox
24		What category of user would you class yourself as?	Likert scale
**Technology section**			
25		A list of 12 potential reasons has been compiled for using Facebook. For each, please state whether you agree, disagree, or it is not applicable to using Facebook.	Table of radio buttons
26		Which of the following factors would discourage you from using Facebook?	Table of radio buttons
27		Do current technologies such as smartphones and tablet PCs encourage you to use Facebook more often?	Radio buttons
**Social impact**			
28		Quality of Life (QoL) is used to evaluate the general well-being of individuals and societies (eg, recreation and leisure time and social belonging). Do you feel using Facebook contributes to your overall QoL?	Radio buttons
29		Do you feel it strengthens the relationship with the people you connect with?	Radio buttons
30		How important do you feel Facebook is for keeping in contact with family and friends?	Likert scale
31		Generally speaking, how do you feel when using Facebook during the following three stages, from feeling very down to feeling very good?	Table of radio buttons
32		You have reached the end of the survey. Please provide any observations/criticisms which you feel may improve future survey takers’ experience.	Textbox
33		If you would like to receive a copy of the analysis, please provide an email address or contact information.	Textbox

### Statistical Analysis

The number of completed surveys is stated along with the responses to each of the 33 questions by each cohort, which are directly contrasted using frequency analysis and horizontal stacked bar charts. Statistical significance between the groups—younger users versus older users—per question was tested using the N-1 chi-square test for comparing independent proportions (*P*<.05).

Multivariate logistic regression was used to assess potential confounders that might bias the hypothesis that Facebook has a positive impact on the QoL for a greater proportion of older users in comparison to younger users. The independent or exposure variables included age group (ie, younger or older cohort), gender, country (ie, 17 different nationalities), computer literacy, and the type of user (eg, frequent user or occasional user). The dependent variable, or response variable, was binary (ie, Facebook has a positive or negative impact, or none at all, on QoL). The model provides odds ratios (ORs) that indicate how each exposure variable contributes to a participant stating that Facebook has an impact on their QoL. The model is described in equation 1:

*logit(p)* = β_0_+ Σ^n^_i=0_β_i_*X*_i_(1)

where β_0_ is the intercept, β_i_ is a vector of coefficients (log odds), and *X*_i_ a vector of values from each independent/exposure variable. All data analysis was carried out using the R programming language in combination with RStudio (RStudio, Boston, MA).

## Results

### Overview

**Figure 1:**
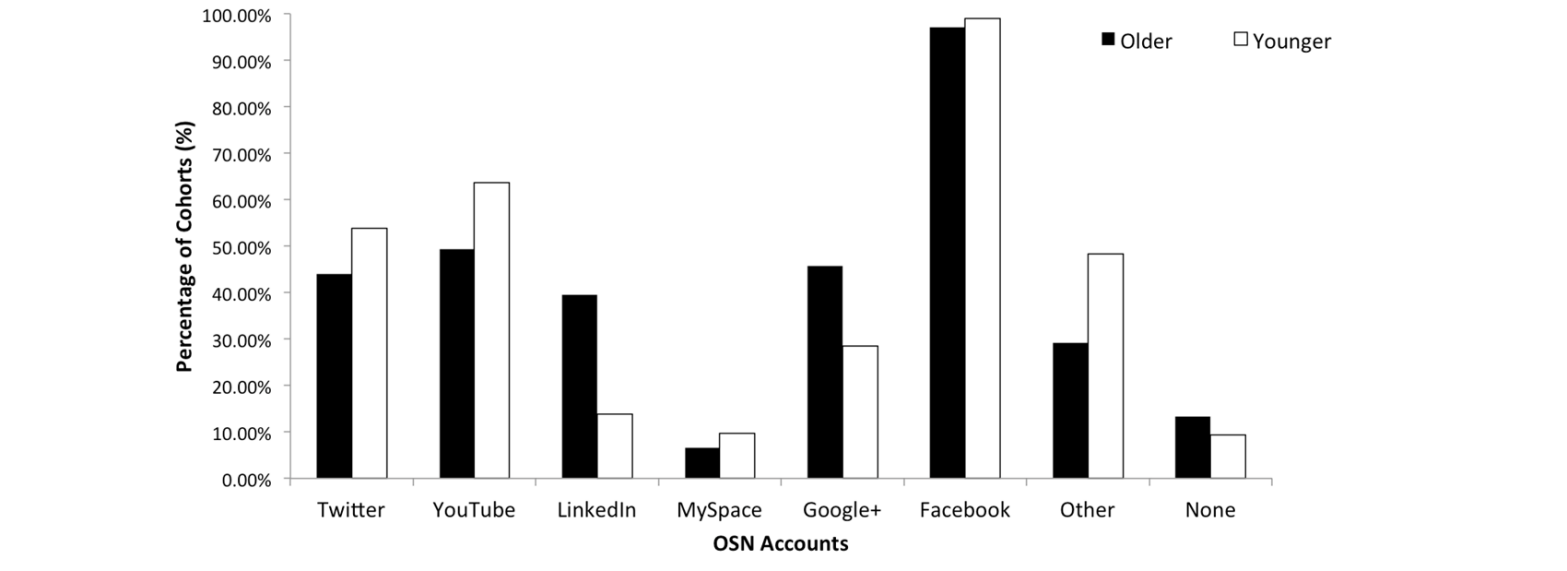
Online social network (OSN) accounts among older and younger users.

Excluding ineligible participants or those with partial surveys, a total of 919 completed surveys were collected, with 446 younger users and 473 older users. Demographics for the two cohorts are provided in Table 2. This table shows an equal distribution of gender in both cohorts, which eliminates gender bias, a common confounding factor. While computer literacy was slightly higher among the younger group, this was to be expected; however, the difference was minimal. Figure 1 shows the popularity of SNS among the cohorts. Younger users preferred Twitter and YouTube whereas older users preferred LinkedIn and Google+.

**Table 2:** Demographics of older and younger participants.

Demographics	Older users (n=473)	Younger users (n=446)
Male, n (%)	173 (36.6)	140 (31.4)
Female, n (%)	300 (63.4)	306 (68.6)
Age in years, mean (SD)	59.35 (6.92)	21.81 (2.48)
Age in years, range	50-87	18-30
Computer literacy^a^, mean (SD)	3.27 (0.84)	3.64 (0.64)

^a^Computer literacy ranges from 1 (novice) to 5 (expert).

### Functional Usage

**Figure 2:**
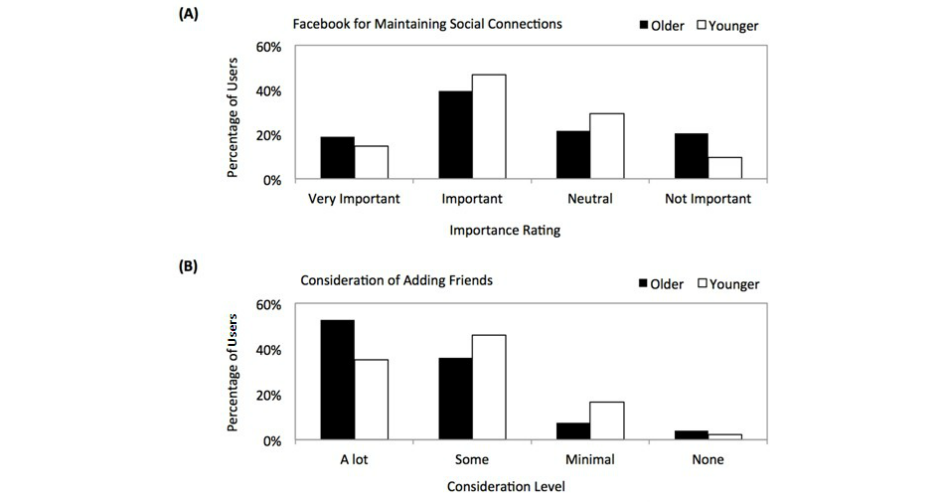
(A) The importance of Facebook for maintaining connections among older and younger users and (B) the consideration taken by both cohorts when adding new friends.

Figure 2 (A) shows how both cohorts considered how important Facebook is for maintaining real-world social connections, and Figure 2 (B) shows how much consideration went into adding or accepting new friends. Results show older users put more consideration into who they connect with; 249 out of 473 (52.6%) older users consider who they connect with *a lot* versus 157 out of 446 (35.2%) younger users consider who they connect with *a lot* (*P*<.001).

**Figure 3:**
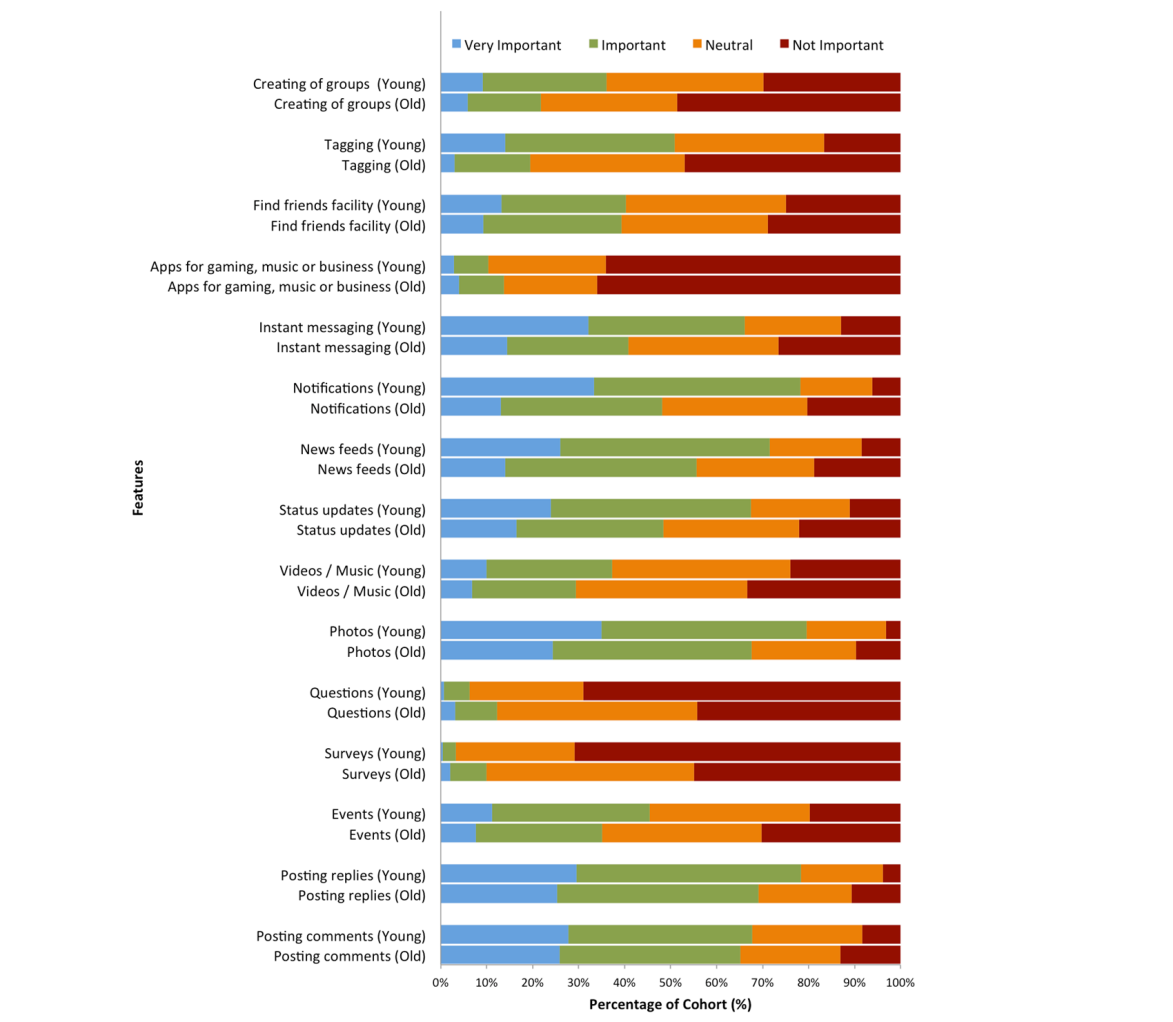
Ratings of Facebook features by older and younger users.

Figure 3 presents the most important Facebook features. Applying statistical significance, younger users gave greater importance to creating groups, tagging, instant messaging, notifications, news feeds, status updates, and photos. However, older users gave greater importance to questions (*P*<.001) and surveys (*P*<.001). In reference to question 10 (see Table 1), only 7.6% (36/473 older and 34/446 younger) of subjects in both cohorts agreed that maintaining an up-to-date Facebook profile (eg, profile picture and relationship status) is *Very Important*. When asked how difficult it is to use Facebook’s features, older users encountered more usability problems (160/473, 33.8%) than younger users (64/446, 14.3%) (*P*<.001). For all users, notifications and news feeds were considered important, but photos and posting were the most important features.

### Patterns of Usage

**Figure 4:**
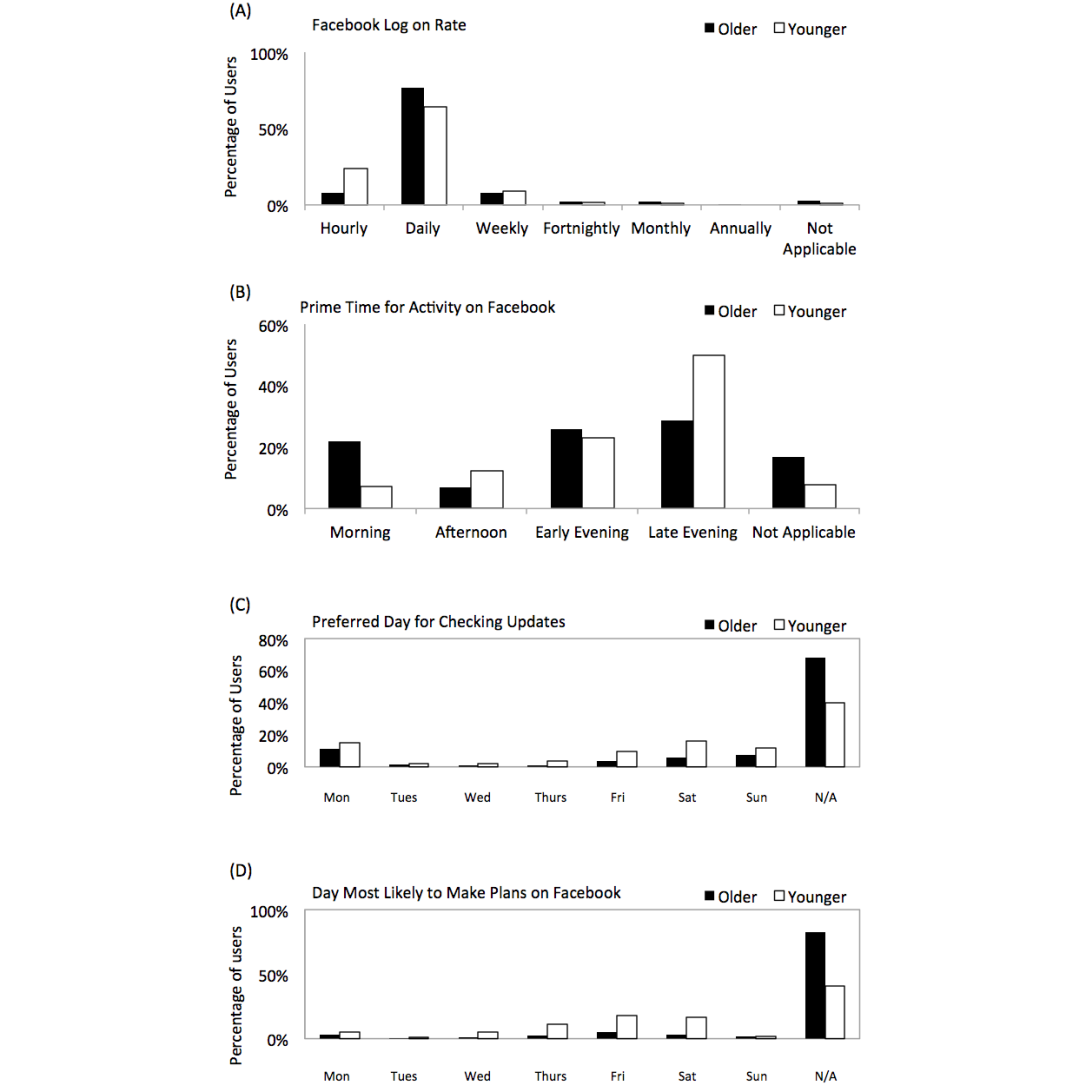
(A) The frequency of how often both cohorts log into Facebook, (B) where each cohort designated their prime time for activity on Facebook, (C) which days the two cohorts are most likely to check Facebook for updates, and (D) which days the cohorts stated to be their most likely to use Facebook for making plans and arranging social events. N/A: not applicable.

Figure 4 shows graphs that indicate when both cohorts are likely to engage with Facebook. Most subjects stated they log on *daily* —362 out of 473 (76.5%) for older users and 286 out of 446 (64.1%) for younger users, as seen in Figure 4 (A). However, more younger users (105/446, 23.5%) log in *hourly* when compared to older users (38/473, 8.0%) (*P*<.001). In addition, more younger users are active in the late evening (135/446, 28.5%) versus in the morning (222/446, 49.8%) (*P*<.001), whereas more older users are active in the morning (103/473, 21.8%) versus in the late evening (32/473, 6.8%) (*P*<.001), as seen in Figure 4 (B). Also, regarding question 22 (see Table 1), 281 of 446 younger users (63.0%) use Facebook during weekdays, whereas the majority of older users (253/473, 53.5%) use Facebook independent of whether it is a weekday or the weekend. Figure 4 (C) indicated that older users do not feel they have a specific day for checking updates. It is also evident from Figure 4 (D) that older people do not use Facebook to make plans or arrange social events, whereas younger users do. In answering question 19 (see Table 1), more younger users ranked Facebook as being *Important* or *Very Important* for planning and broadcasting events (206/446, 46.2%) versus 274 of 473 older users (57.9%) (*P*<.001).

**Figure 5:**
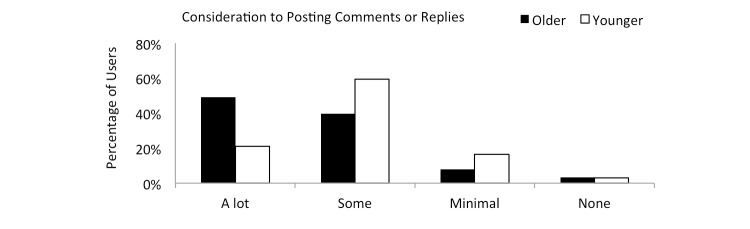
Amount of consideration given to posting comments or a reply for both cohorts.

Figure 5 shows the amount of consideration given to posting comments to a friend. A higher percentage of older subjects (232/473, 49.0%) provide *a lot* of consideration to posting comments compared to the younger cohort (94/446, 21.1%) (*P*<.001).

**Figure 6:**
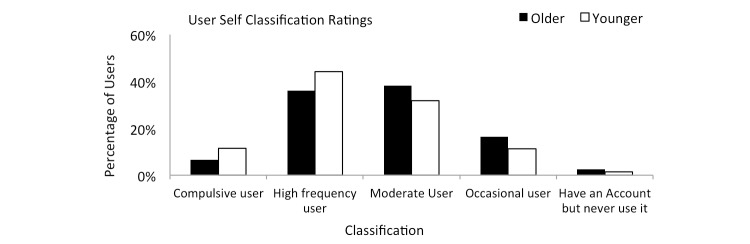
Behavior classification in both cohorts.

Answers to question 20 from Table 1 (see Table 3) asked users to consider their overall behavior and then to rank it within three options— *Responder*, *Observer*, or *Instigator*. Both cohorts primarily declared their behavior to be *Respond to others*. However, younger users ranked this higher when compared to older users. Answers to question 24 from Table 1 (see Figure 6) show that the majority of users in both cohorts classify themselves as a *high frequency user* or a *moderate user*.

**Table 3:** Behavior rankings.

Descriptor	Older users		Younger users	
	Total score^a^	Overall rank^a^	Total score	Overall rank
Respond to others’ activity	859	1	936	1
Observe others' activity	685	2	800	2
Instigate activity (eg, posting comments, videos, and pictures)	636	3	673	3

^a^Score is a weighted calculation. Items ranked first are valued higher than the following items; the score is the sum of all weighted rank counts.

### Technology

**Figure 7:**
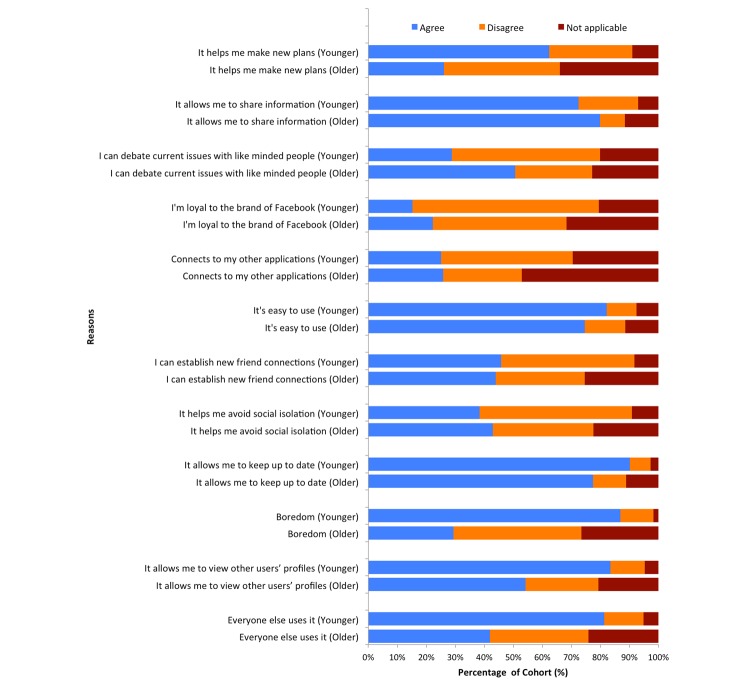
A list of 12 reasons for using Facebook according to both cohorts.

Figure 7 shows 12 reasons for using Facebook. The main reasons for the younger cohort to use Facebook, that are all statistically significant (*P*<.001) in comparison to older users, is that it helps them in making new plans (278/446, 62.3% younger vs 123/473, 26.0% older), they can view other profiles (372/446, 83.4% younger vs 256/473, 54.1% older), and the fact that everybody else uses it (363/446, 81.4% younger vs 198/473, 41.9% older). Interestingly, 387 of 446 younger users (86.8%) engage with Facebook due to boredom, which is in contrast to 139 of 473 older users (29.4%) who do so. Conversely, a greater proportion of older users identified that they engage with Facebook because they can debate with like-minded people, which is distinct from the younger cohort (239/473, 50.5% older vs 128/446, 28.7% younger).

**Figure 8:**
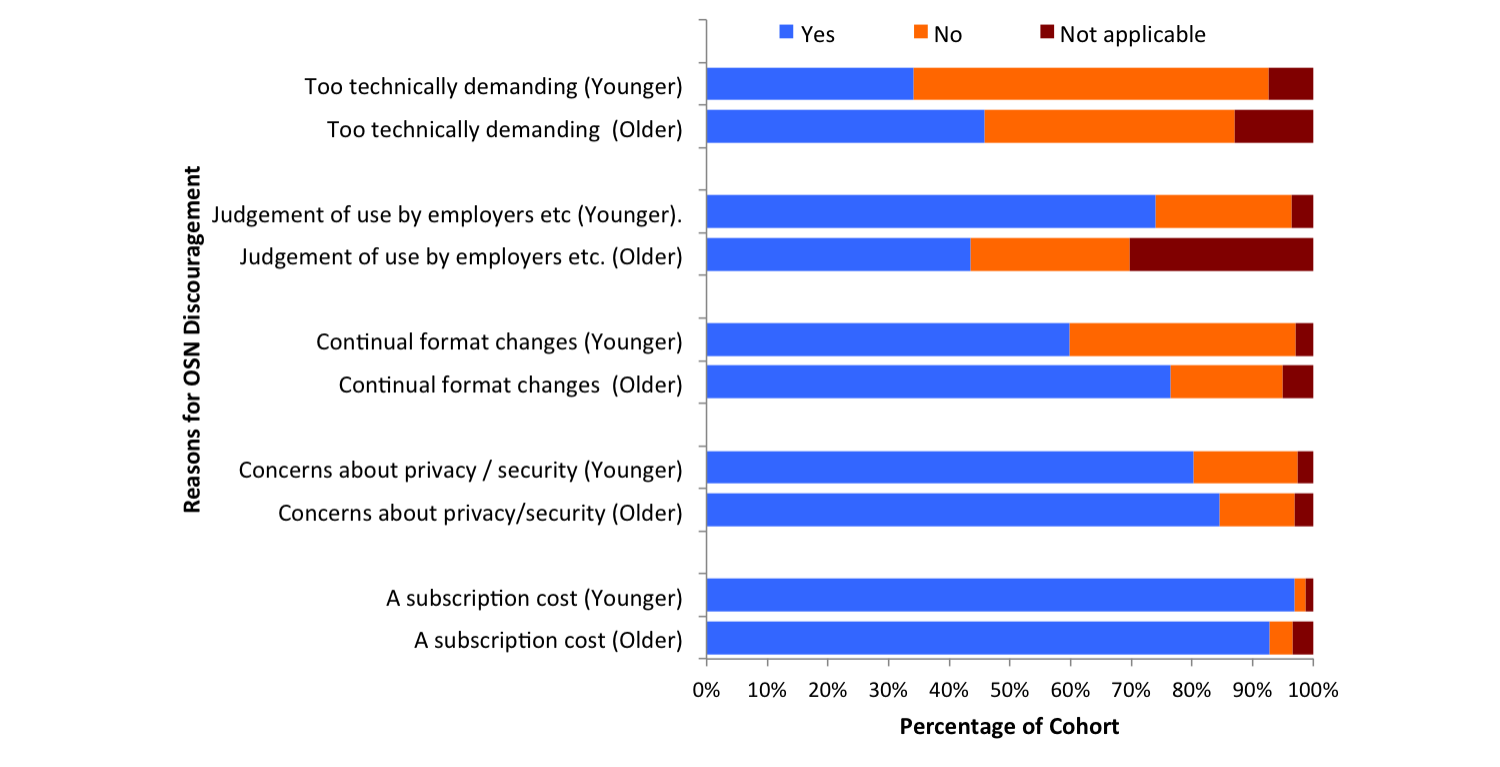
Factors that discourage the use of Facebook for younger and older users. OSN: online social networks.

Figure 8 shows the factors that could discourage users from using Facebook. A greater percentage of older users agree that such factors would include the following: (1) Facebook is too technically demanding (217/473, 45.9% older vs 152/446, 34.1% younger) (*P*<.001) and (2) the continual format changes would discourage users from engaging (362/473, 76.5% older vs 267/446, 59.9% younger) (*P*<.001). Conversely, a greater percentage of younger users agree that profile viewing from potential employers is a key reason not to engage (330/446, 74.0% younger vs 206/473, 43.6% older) (*P*<.001). Interestingly, 354 of 446 younger users (79.4%) agreed that technologies such as mobile phones and tablet personal computers (PCs) encourage them to use Facebook more frequently, whereas only 146 of 473 older users (30.9%) agreed with this statement (*P*<.001).

### Social Impact

**Figure 9:**
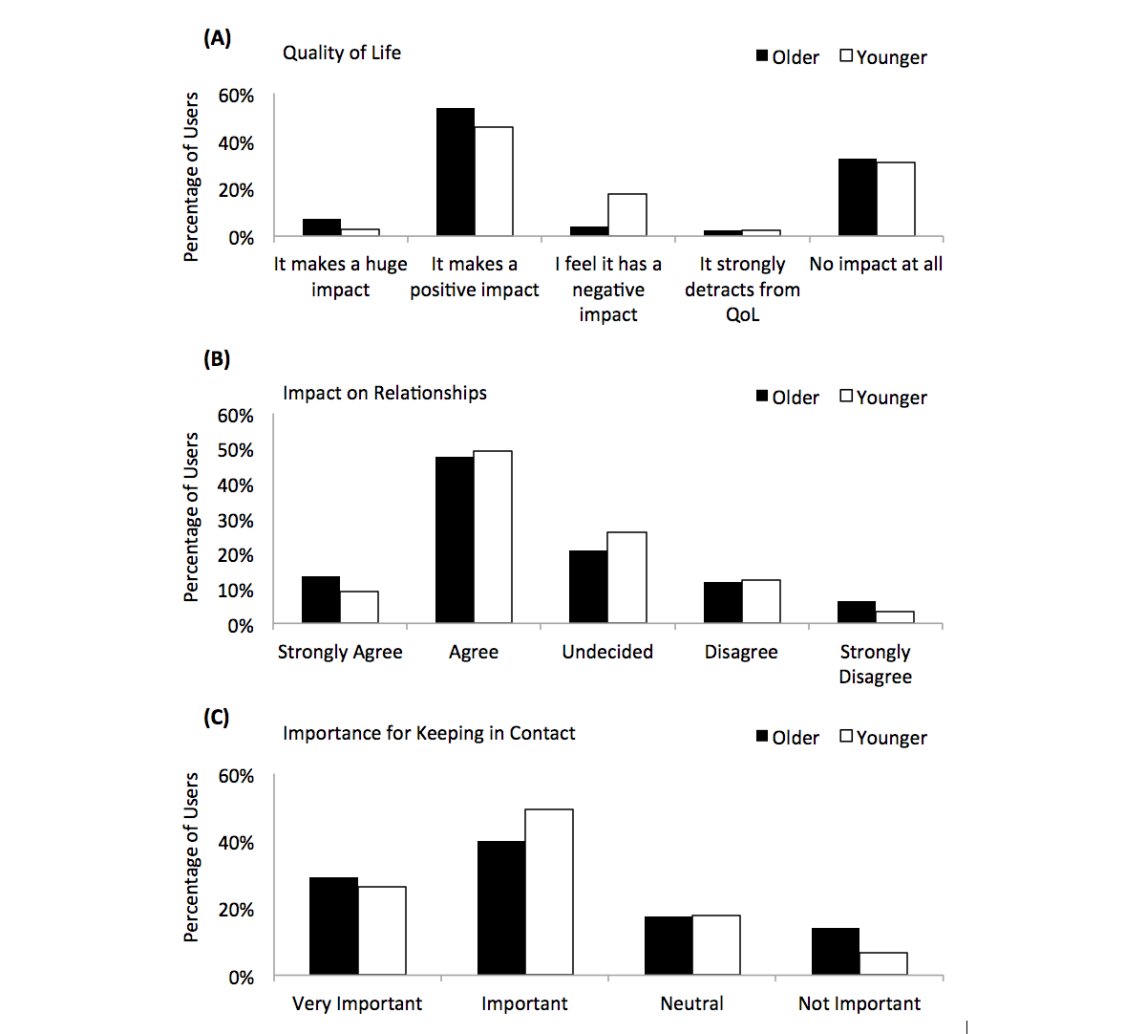
(A) Both cohorts feel Facebook contributes to their quality of life (QoL), (B) users feel it strengthens the relationship with the people they are connected to, and (C) users feel Facebook is important for keeping in contact with family/friends.

Figure 9 presents graphs that show how Facebook contributes to the users’ QoL and their relationships with people. In comparison to younger users, a greater proportion of older users (289/473, 61.1% older vs 218/446, 48.9% younger) (*P*<.001) stated that Facebook has either a *positive impact* or a *huge impact* on their QoL, as seen in Figure 9 (A). There are few differences between the cohorts in Figure 9, (B) and (C). However, a greater percentage of older users *strongly agree* that Facebook strengthens their relationships with other people (64/473, 13.5% older vs 40/446, 9.0% younger) (*P*=.02).

Table 4 indicates the ORs for each exposure variable that may contribute to a participant indicating that Facebook contributes to their QoL. The table confirms that younger users are less likely (OR 0.45, *P*<.001) to indicate that Facebook improves their QoL. Conversely, there are greater odds that Facebook will have a positive impact on the QoL of older users when compared to younger users. However, Table 4 indicates that there are two other statistically significant ORs related to the frequency of using Facebook. Thus, those participants who ranked themselves as a *moderate* or *occasional* user were less likely to state that Facebook has an impact on their QoL. However, on inspection, this is not a confounding factor since the variable is considerably proportionately split between the younger and older cohort (ie, 26% of younger users are moderate/occasional users who said Facebook has no positive impact on QoL and, similarly, 29% of older users are moderate/occasional users who said Facebook has no positive impact on QoL). While 17 countries were represented in the dataset, this variable was also not confounding. Being German was almost significant (OR 9.25, *P*=.08); however, the confidence interval has a significant range and there were only 6 German participants: an equal split of 3 German participants in each group. Interestingly, the per unit increase in computer literacy, which could be associated with education and socioeconomic status, was not statistically significant (OR 0.92, *P*=.46) in contributing to stating whether Facebook has an impact on QoL.

**Table 4:** Odds ratios for each of the independent (exposure) variables where the response variable is whether Facebook has or has not made an impact on the user’s quality of life.

Exposure variable	Odds ratio	95% CI	SE	*Z*	*P*
Age group (younger)	0.45	0.32-0.62	0.17	-4.74	<.001
Gender (male)	1.19	0.87-1.65	0.17	1.07	.28
Computer literacy (per unit increase)	0.92	0.74-1.14	0.11	-0.75	.46
Country (Germany)	9.25	0.96-22.04	1.29	1.73	.08
Type of user (high-frequency user)	1.12	0.66-1.88	0.27	0.43	.67
Type of user (moderate user)	0.55	0.32-0.93	0.27	-2.20	.02
Type of user (occasional user)	0.05	0.02-0.09	0.38	-8.04	<.001

**Figure 10:**
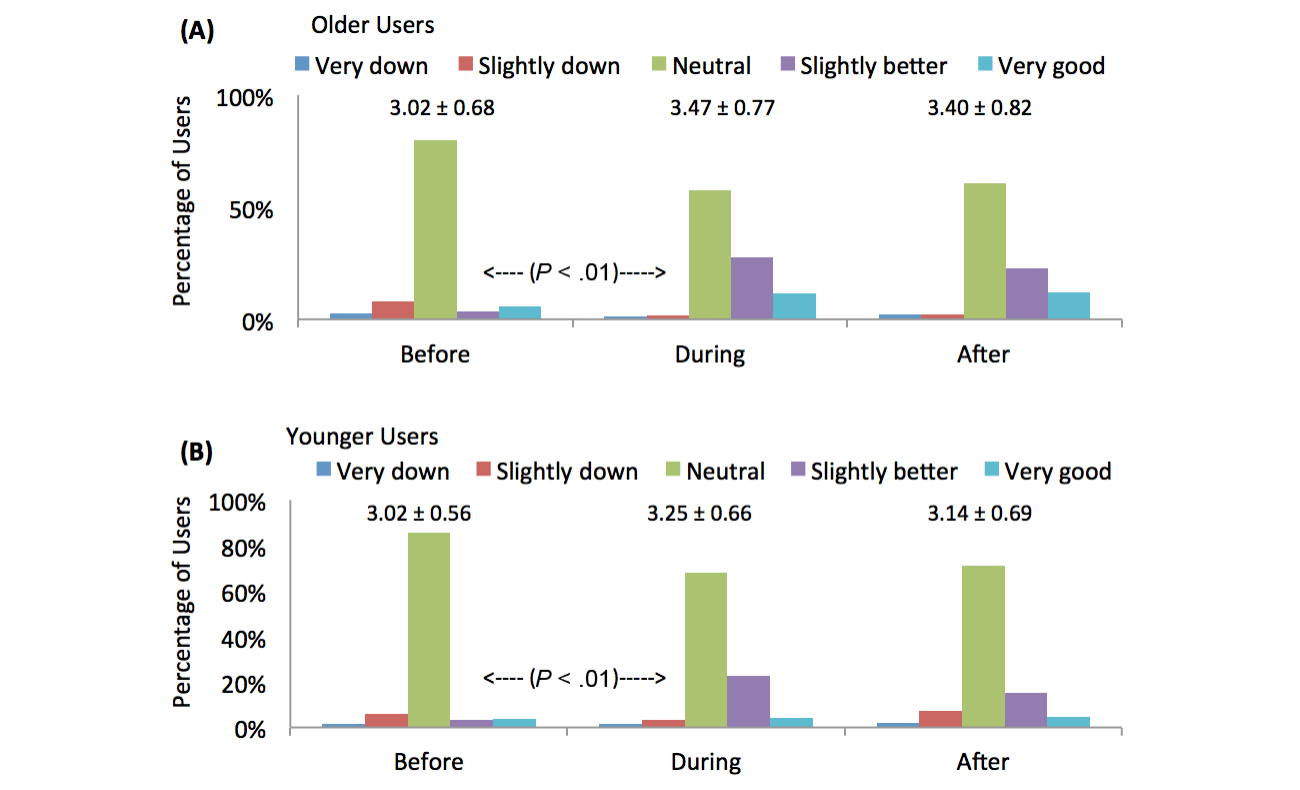
Emotional state before, during, and after using Facebook for (A) older users and (B) younger users. The graphs also present the means and standard deviations for the ratings of the state in each phase (where 1=very down and 5=very good). The P values compare the proportion of users who feel slightly better or very good before and during Facebook engagement for both cohorts.

Figure 10 shows two graphs that illustrate the changes in positive emotion before, during, and after using Facebook. Before using Facebook, 45 of 473 older users (9.5%) felt *good* or *very good*. However, when engaged with Facebook, this statistic increased to 186 out of 473 (39.3%) (*P*<.001). Likewise, before using Facebook, 31 of 446 younger users (7.0%) felt *good* or *very good*, however, when engaged with Facebook this statistic increased to 120 out of 446 (26.9%) (*P*<.001). This also indicates that in comparison to younger users, a greater proportion of older users have more positive emotions—classified as *slightly better* or *very good* —during their engagement with Facebook (186/473, 39.3% older vs 120/446, 26.9% younger) (*P*<.001).

## Discussion

The aim of this study was to disclose the impact of online social networking on QoL, examining the perceptions of users as they engage. The hypothesis was that SNS positively contribute to the QoL of older people. Results disclosed why younger users have 11 times more Facebook “friends” (observed by Quinn et al [7]). This is due to the fact that younger users create new linkages without much consideration. Relating to functional usage, a hypothesis was proposed that anomalies, identified by Quinn et al [8], were due to the fact that each cohort attached different values to different functionalities. Results of this study reveal that differing Facebook features are clearly identifiable in terms of their importance for each of the age groups. In asking about the *prime time* for activity, interpretation of results leads to the conclusion that younger users integrate SNS as a part of their daily life. For these users, online social networking activity occupies a dedicated time within each day, which has been clearly evidenced by the volume of users recorded in this study. Facebook is now an accepted communication modality adjudged to be part of the user’s routine. In this regard, younger users gave greater importance for using SNS for planning and broadcasting new events. However, the view of older users was more divided, with only a slim majority recording *Neutral*, followed by *Important* (171/473, 36.2% and 156/473, 33.0%, respectively). Results therefore disclose older users to be the more reflective users.

Questions concerning patterns of usage evaluated if users had a bias for *Weekday* or *Weekend* usage, following the user metric results contained within Quinn et al [7]. Results demonstrated a strong preference for weekend use among younger respondents when compared to older users. Twice as many older users selected *Weekday* as their preference. Results indicated that given that the majority of younger users were attending university, they accessed their accounts during weekdays. Older users are not bound by such restrictions and results are therefore reflective of such facts. Based on this new evidence for both cohorts, Facebook is now shown to be important for maintaining real-world connections. Results demonstrated that age cohorts are identifiable with particular functionalities. Discouraging factors united users in opposing subscription costs, with concerns relating to privacy/security, and continual platform changes. SNS were shown to strengthen relations, regardless of age, as both cohorts agreed upon its importance for keeping in contact with family and friends. In terms of emotional state, a definite shift was observed when a greater volume of users were recorded among the more positive emotions *During* and *After* usage. It is now shown that usage directly affects the emotional state of users as they engage, the limitation being that emotional state was self-reported. Given the content that users will frequently observe, such as pictures of friends and family or messages from friends, it is often emotive content that will directly stimulate the emotions of a user. As a social platform, it is clearly an established mode of communication. However, results demonstrated no loyalty to the brand, indicating a willingness by both cohorts to potentially switch to an alternative.

Given that older users are engaging extensively in online social media [38], a key aim of this study was to investigate the impact of such users who interact with a social networking application, namely Facebook. A particular focus was to establish what, if any, relationship existed between using social networking technologies and their impact upon users’ QoL. Although a number of works have emerged in the area, it was identified that (1) results were the subject of conjecture and (2) no works addressed the real-world impact upon the QoL of end users and, more specifically, the impact upon older users. This is important since social technology has the potential to alleviate the burden of social isolation. Although generic traits could be shared across both cohorts, there were many characteristics which were identifiable to specific age ranges, as described in Textbox 1.

**Evidence-based personas of older and younger users.**

Younger persona

- They create connections with less consideration, largely due to their real-world social structures; subsequently, they will update profiles frequently due to their life updates (eg, relationships, jobs, and attainment).
- They engage very frequently, due to the fact that the majority of their friends use the same application and the source, therefore, of the majority of social content; subsequently, they check for updates more frequently.
- They prefer weekday activity.
- They provide dedicated time to keep up-to-date with their peers and the news, which is facilitated by the fact that they find the interface easy to use.
- They frequently share information on social network sites (SNS) for making plans.
- They feel that the SNS positively impacts on their quality of life (QoL).

Older persona

- They give a lot of consideration to creating new connections and crucially operate a quality approach to posting; with fewer connections, there are fewer reasons to engage.
- They are more purposeful in their reason for engagement; they only log on for a direct purpose.
- As a cohort, their failure to continue engaging is explicable due to difficulties in using features on a multifaceted platform.
- Older users encounter more usability problems with the user interface.
- Contemporary technologies do not encourage older users to engage, most likely due to the low adoption rates of other technologies.
- They feel that the SNS positively impacts on their QoL.

In conjunction with previous research, results from 919 surveyed users—446 younger users (18-25 years) and 473 older users (50+ years)—form a new body of knowledge applicable to many domains, from policy makers to SNS designers. Results showed that older users have a quality, rather than a quantity, approach to SNS usage. This was directly in contrast to that of younger users. Although older users interacted with the SNS less frequently, they gained a significantly greater QoL and emotional benefit to using Facebook when compared to younger users. Future work could explore how SNS effectiveness can help users avoid social isolation.

## Appendix

Multimedia Appendix 1 Research questions.

Multimedia Appendix 2 Older people’s organizations.

## Abbreviations

LSI: Life Satisfaction Index
OR: odds ratio
QoL: quality of life
SNS: social network site(s)
