# Supplementary material for: Exploring the Relationship Between Online Social Network Site Usage and the Impact on Quality of Life for Older and Younger Users: An Interaction Analysis
Source: J Med Internet Res. 2016 Sep 29;18(9):e245. doi: 10.2196/jmir.5377 (PMC5064125; doi:10.2196/jmir.5377)
Supplement: Multimedia Appendix 1 [file jmir_v18i9e245_app1.pdf]

Appendix 1

| *Number* | *Functional Usage* |
|---|---|
| 1 | Why do younger users have so many more online friends? |
| 2 | Why does the functional usage of OSN vary with age? |
| 3 | Why does the rate at which younger and older users maintain their profile vary so markedly? |
| 4 | Why do older users fail to continue engaging after they create an account? |
| 5 | Why do older users engage less frequently? |
| 6 | Why do young users engage more frequently? |
| | ***Behavioural Research Question*** |
| 7 | Why is the engagement of older users more sporadic than that of younger users? |
| 8 | Regardless of age, why does activity peak between 10-11pm? |
| 9 | Why do varying age cohorts peak in activity on different days? |
| 10 | Does a correlation exist between online activity and real world activity? |
| 11 | Do younger users have a more liberal attitude to using OSN as a modality for communication? |
| | ***Technology Focused Research Questions*** |
| 12 | What motivates users to engage with OSN? |
| 13 | Which factors discourage use? |
| 14 | Do mobile technologies (smartphones etc.) encourage use? |
| | ***Quality of Life Research Questions*** |
| 15 | Do OSN contribute to your QoL? |

| | |
|---|---|
| 16 | Do OSN strengthen relationships? |
| 17 | How important are OSN for keeping in contact with family and friends? |
| 18 | Does usage vary with mood? |
