# Supplementary material for: Exploring the Relationship Between Online Social Network Site Usage and the Impact on Quality of Life for Older and Younger Users: An Interaction Analysis
Source: J Med Internet Res. 2016 Sep 29;18(9):e245. doi: 10.2196/jmir.5377 (PMC5064125; doi:10.2196/jmir.5377)
Supplement: Multimedia Appendix 2 [file jmir_v18i9e245_app2.pdf]

Appendix 2 Older People Organisations

Organisation Name	Web Address
Age Scotland	http://www.ageuk.org.uk/scotland/
Age Northern Ireland	http://www.ageuk.org.uk/northern-ireland/
Age Wales	http://www.ageuk.org.uk/cymru/
Age Action Ireland	http://www.ageaction.ie/
Gransnet	http://www.gransnet.com/
Go-on-uk	http://www.go-on.co.uk/
Joseph Rowntree Foundation	http://www.jrf.org.uk/
New Dynamics of Ageing Programme	http://www.newdynamics.group.shef.ac.uk
Facebook advertising	https://www.facebook.com/advertising
